# Editorial: Coding and non-coding RNA-based complexes in organismal development and disease pathogenesis

**DOI:** 10.3389/fgene.2022.1032665

**Published:** 2022-09-16

**Authors:** Pascal Chartrand, Chiara Gamberi

**Affiliations:** ^1^ Department of Biochemistry and Molecular Medicine, Université de Montréal, Montréal, QC, Canada; ^2^ Department of Biology, Coastal Carolina University, Conway, SC, United States

**Keywords:** RNA regulation, disease, development, coding RNAs, non-coding RNAs

Progress in “omics” technologies indicate that most of the human genome is transcribed at some point during development. While the importance of protein-coding mRNAs remains of primary interest, the majority of the cellular RNAs appear to be non-coding and subject to regulation (ENCODE Project Consortium, 2012; Hon et al., 2017). Non-coding RNAs could potentially have direct regulatory functions or affect gene expression through “noise”. One open question remains how to connect the wealth of molecular detail with phenotypic effects such as changes in physiological function and adaptation. The articles included in this Research Topic explore fundamental, developmental and pathological processes that involve coding and non-coding RNAs. This series of articles constitute the second volume of a Research Topic on RNA in Development and Disease previously published in Frontiers in Genetics in 2020 (Chartrand et al., 2020). While the first volume focused on mechanisms of post-transcriptional gene regulation such as mRNA export, translational control and mRNA localization, this second volume covers new ground as it includes non-coding RNAs and mechanisms not previously discussed, such as alternative splicing and mRNA decay.

A first group of articles focus on the evolution and function of non-coding RNAs. On the origin and evolution of non-coding RNAs, Palazzo and Kejiou offer a global view on non-darwinian evolution and describe how sloppy and inefficient processes within cells may explain how junk RNA can evolve toward functional non-coding RNA. Long non-coding RNAs have emerged as key regulators of gene expression, particularly due to their capacity to bind both protein cofactors (transcriptional regulators or DNA modifiers) and interact with DNA with high sequence specificity. In their article Alecki and Vera review the different non-canonical nucleic acid structures formed by non-coding RNA and DNA, focusing on R-loops and DNA:RNA triplexes. The authors also critically discuss the various techniques used to study the non-canonical nucleic structures formed by non-coding RNAs. Small non-coding RNAs also participate in epigenetic silencing, translational repression and transcript degradation. One group of small non-coding RNAs called PIWI-interacting RNAs (piRNAs) plays key roles in the repression of transposons in the ovary and testis, while their role in somatic tissues has been more controversial. Interestingly, Tsuji et al. now report the expression of piRNAs in the intersegmental muscles of the tobacco hawkmoth, where these small RNAs are involved in programmed cell death during muscle atrophy.

A second group of articles review key mechanisms involved in the post-transcriptional regulation of gene expression. Alternative splicing plays a fundamental role in this regulation since, by choosing which exons are maintained or excluded in a given mRNA, it increases the complexity of both transcriptome and proteome in a tissue-specific manner. In their review, Titus et al. explore the different functions of protein-coding spliceforms in development and diseases. Their review particularly highlights the role of alternative splicing in the regulation of gene expression via its coupling with nonsense mediated mRNA decay and mRNA localization.

Localized translation contributes to the spatial regulation of protein synthesis by targeting transcripts to specific subcellular compartments. A key player in this process is the Signal Recognition Particle (SRP), a ribonucleoprotein complex involved in the co-translational targeting of mRNAs to the endoplasmic reticulum (ER) and in the protection of these transcripts from degradation. Kellogg et al. review the role of the SRP and describe how mutations in SRP subunits can lead to specific pathologies such as hematological disease, autoimmunity, neurological diseases and cancer.

Finally, the cellular functions of both coding and non-coding RNAs must be controlled in a timely fashion, which implies the degradation of these molecules at some point. Removal of the 7-methyl guanosine cap is one of the key steps initiating the degradation of capped mRNA in eukaryotes. In their review, Vidya and Duchaine extensively describe the mechanisms regulating the activation of mRNA decapping in various organisms and how cytoplasmic P bodies participate in the regulation of decapping factors.

Since the first predictions that RNA may have regulatory functions over 50 years ago (Jacob and Monod, 1961; Britten and Davidson, 1969) a great deal has been learnt about non-coding ribosomal, transfer, spliceosomal and nucleolar RNAs. This Research Topic captures exemplary cases of coding and non-coding RNA regulation. It is well-accepted that modifications in protein sequence and/or translation levels may have phenotypic consequences. Increasingly more appreciated is the potential for sequence changes in the non-coding regions to bear functional consequences. Notably, single nucleotide polymorphisms linked to disease are found largely in non-coding genomic regions (Hindorff et al., 2009; Ricaño-Ponce and Wijmenga, 2013) where they could influence the activity of promoters, enhancers, and epigenetic control. Such changes might as well affect several levels of post-transcriptional events e.g., alternative splicing, RNA folding and processing, microRNA binding and, potentially, the expression of quantitative traits yielding “individualized” outputs underlying the observed phenotypic variation and disease susceptibility. As RNA biologists strive to integrate big data with refined knowledge of the precise workings of specific pathways, it has become clear that RNA regulation is central to cellular and organismal function and may influence biological diversity and disease.
